# Supplementary material for: Modelling Skylarks (Alauda arvensis) to Predict Impacts of Changes in Land Management and Policy: Development and Testing of an Agent-Based Model
Source: PLoS One. 2013 Jun 6;8(6):e65803. doi: 10.1371/journal.pone.0065803 (PMC3675089; doi:10.1371/journal.pone.0065803)
Supplement: Supporting Information S4 — The skylark ODdox as a zipped archive. (ZIP) [file pone.0065803.s004.zip › Skylark_ODdox/functions_0x66.html]

ALMaSS Skylark ODdox: Class Members


ALMaSS Skylark ODdox
 2.0


- Main Page
- Related Pages
- Classes
- Files

- Class List
- Class Index
- Class Hierarchy
- Class Members

- All
- Functions
- Variables
- Typedefs

- a
- b
- c
- d
- e
- f
- g
- h
- i
- j
- k
- l
- m
- n
- o
- p
- r
- s
- t
- u
- v
- w
- x
- y
- z
- ~

Here is a list of all class members with links to the classes they belong to:

### - f -

- F\_Mig\_Mort
  : Skylark\_Population\_Manager
- F\_Owner
  : skTTerritory
- FA\_AmmoniumSulphate()
  : Farm
- FA\_GreenManure()
  : Farm
- FA\_Manure()
  : Farm
- FA\_NPK()
  : Farm
- FA\_PK()
  : Farm
- FA\_Sludge()
  : Farm
- FA\_Slurry()
  : Farm
- Farm()
  : Farm
- FarmEvent()
  : FarmEvent
- FarmManagement()
  : FarmManager
- FarmManager()
  : FarmManager
- FeedYoung()
  : Skylark\_Female
- FemaleOccupy()
  : SkTerritories
- Field()
  : Field
- FieldBoundary()
  : FieldBoundary
- FileAppendOutput()
  : probe\_data
- FileOutput()
  : probe\_data
- FillVegAreaData()
  : Landscape
- FindClosest()
  : Population\_Manager
- FindCropNum()
  : CropData
- FindDiff()
  : CropData
- FindFieldCenter()
  : Landscape
- FindLongestAxis()
  : Landscape
- FindValidXY()
  : Landscape
- FledgelingProbeOutput()
  : Population\_Manager
  , Skylark\_Population\_Manager
- ForceArea()
  : Landscape
- ForceGrowthDevelopment()
  : VegElement
  , LE
- ForceGrowthInitialize()
  : LE
  , VegElement
- ForceGrowthSpringTest()
  : VegElement
- ForceGrowthTest()
  : LE
  , VegElement
- ForestElement()
  : ForestElement
- found
  : skSpiralResult
- FP\_GreenManure()
  : Farm
- FP\_LiquidNH3()
  : Farm
- FP\_ManganeseSulphate()
  : Farm
- FP\_Manure()
  : Farm
- FP\_NPK()
  : Farm
- FP\_NPKS()
  : Farm
- FP\_PK()
  : Farm
- FP\_Sludge()
  : Farm
- FP\_Slurry()
  : Farm
- Freshwater()
  : Freshwater
- FungicideTreat()
  : Farm


- Generated on Thu Jan 10 2013 13:15:39 for ALMaSS Skylark ODdox by
   1.8.1.1
